# Supplementary material for: Regulatory properties of transcription factors with diverse mechanistic function
Source: PLoS Comput Biol. 2024 Jun 10;20(6):e1012194. doi: 10.1371/journal.pcbi.1012194 (PMC11192337; doi:10.1371/journal.pcbi.1012194)
Supplement: S1 Text — (I). Model and simulation methodology. (II) Derivation of Fold-change from Thermodynamic model. (III) Fitting model to pre-existing data. (IV) Noise in gene expression. (V) Autoregulating gene. (PDF) [file pcbi.1012194.s001.pdf]

Supplementary Information for Regulatory properties of transcription factors with diverse mechanistic function

Md Zulfikar Ali,[1,2,3] Sunil Guharajan,[1,2] Vinuselvi Parisutham,[1,2] and Robert C. Brewster[1,2]

[1]*Department of Systems Biology, University of Massachusetts Medical School, 368 Plantation St, Worcester, MA 01605*

[2]*Department of Microbiology and Physiological Systems, University of Massachusetts Medical School, 368 Plantation St, Worcester, MA 01605*

[3]*Department of Geology, Physics and Environmental Science, University of Southern Indiana, 8600 University Blvd, Evansville, IN 47712*

I. MODEL AND SIMULATION METHODOLOGY

The full kinetic model of gene regulation by a single TF is shown in Fig 1B. For simplicity we assume that there is only one binding site for TF and one for the polymerase. The model accounts for binding of TF and polymerase independently at rates that are proportional to the free TF ($N_{\rm TF}$) or polymerase concentration ($N_{\rm P}$) and their corresponding binding rates $k_{\rm on,TF}$ and $k_{\rm on,P}$. The TF and the polymerase unbind/dissociate from the bound states at the rate that is independent of the TF/polymerase concentration and is only dependent on the interaction between the TF and the polymerase, and binding site identity. We denote the unbinding rates of the TF and polymerase as $k_{\rm off,TF}$ and $k_{\rm off,P}$, respectively. The polymerase may also unbind through a productive initiation event where it will create an mRNA/protein. The regulatory role of the TF is encoded in two ways. The first, which we call stabilization, is represented as a constant factor, β, that alters the rate of TF and polymerase dissociation when co-bound, i.e., the unbinding rates of TF and polymerase from the co-bound states are altered to $\beta^{-1}k_{\rm off,TF}$ and $\beta^{-1}k_{\rm off,P}$. The second regulatory mechanism, acceleration (α), is a constant multiplicative factor that modulates the initiation rate, r. These factors fall in the range between 0 and ∞; values greater than 1 represent regulatory interactions that promotes gene expression (faster initiation or greater polymerase occupancy at the promoter), while values less than 1 represent regulatory interactions that repress expression (slower initiation or lower polymerase occupancy at the promoter. For simplicity and keeping the model tractable, we assume that the mRNA and protein production rates are incorporated in the initiation rate r and we do not include mRNA dynamics explicitly in the model. Finally, the functional product of the gene m is "degraded" through dilution due to growth, at the rate γ which is set by the growth rate.

The chemical master equations (CMEs) governing the dynamics of gene expression (m) can then be given by the following equations,

$$\begin{aligned}
\frac{d}{dt}P_{00}(m) &= -(N_{\rm TF}k_{\rm on,TF}+N_{\rm P}k_{\rm on,P})P_{00}(m)+k_{\rm off,TF}P_{01}(m)+k_{\rm off,P}P_{10}(m)+rP_{10}(m-1)-\gamma mP_{00}(m)\\
&\quad +\gamma(m+1)P_{00}(m+1), &(1)\\
\frac{d}{dt}P_{01}(m) &= N_{\rm TF}k_{\rm on,TF}P_{00}(m)-(k_{\rm off,TF}+N_{\rm p}k_{\rm on,P})P_{01}(m)+\beta^{-1}k_{\rm off,P}P_{11}(m)+\alpha rP_{11}(m-1)-\gamma mP_{01}(m)\\
&\quad +\gamma(m+1)P_{01}(m+1), &(2)\\
\frac{d}{dt}P_{10}(m) &= N_{\rm P}k_{\rm on,P}P_{00}(m)-(k_{\rm off,P}+N_{\rm TF}k_{\rm on,TF})P_{10}(m)+\beta^{-1}k_{\rm off,TF}P_{11}(m)-rP_{10}(m)-\gamma mP_{10}(m)\\
&\quad +\gamma(m+1)P_{10}(m+1), &(3)\\
\frac{d}{dt}P_{11}(m) &= N_{\rm P}k_{\rm on,P}P_{01}(m)+N_{\rm TF}k_{\rm on,TF}P_{10}(m)-\beta^{-1}(k_{\rm off,P}+k_{\rm off,TF})P_{11}(m)-\alpha rP_{11}(m)-\gamma mP_{11}(m)\\
&\quad +\gamma(m+1)P_{11}(m+1). &(4)
\end{aligned}$$

Here, $P_{i,j}(m)$ denotes the probability of having m proteins at time t in the state (i,j). i and j denotes the occupancy of polymerase and TF respectively and 0(or 1) indicates if the binding site is occupied (or free). Summing both side of the equations from $m=0$ to $m=\infty$, we obtain the rate equation for mean occupancy in each state

$$\begin{aligned}
\frac{d}{dt}\langle P\rangle_{00} &= -(N_{\rm TF}k_{\rm on,TF}+N_{\rm P}k_{\rm on,P})\langle P\rangle_{00}+k_{\rm off,TF}\langle P\rangle_{01}+(k_{\rm off,P}+r)\langle P\rangle_{10}, &(5)\\
\frac{d}{dt}\langle P\rangle_{01} &= N_{\rm TF}k_{\rm on,TF}\langle P\rangle_{00}-(k_{\rm off,TF}+N_{\rm P}k_{\rm on,P})\langle P\rangle_{01}+(\beta^{-1}k_{\rm off,P}+\alpha r)\langle P\rangle_{11}, &(6)\\
\frac{d}{dt}\langle P\rangle_{10} &= N_{\rm P}k_{\rm on,P}\langle P\rangle_{00}-(N_{\rm TF}k_{\rm on,TF}+k_{\rm off,P}+r)\langle P\rangle_{10}+\beta^{-1}k_{\rm off,TF}\langle P\rangle_{11}, &(7)\\
\frac{d}{dt}\langle P\rangle_{11} &= N_{\rm P}k_{\rm on,P}\langle P\rangle_{01}+N_{\rm TF}k_{\rm on,TF}\langle P\rangle_{10}-\big(\beta^{-1}(k_{\rm off,P}+k_{TF})+\alpha r\big)\langle P\rangle_{11}, &(8)\\
&&(9)
\end{aligned}$$

Similarly, multiplying both side of Eqns. A(1-4) by m and summing over $m=0$ to $m=\infty$ give

$$\begin{aligned}
\frac{d}{dt}\langle m\rangle_{00} &= -(N_{\rm TF}k_{\rm on,TF}+N_{\rm p}k_{\rm on,P}+\gamma)\langle m\rangle_{00}+k_{\rm off,TF}\langle m\rangle_{01}+(k_{\rm off,P}+r)\langle m\rangle_{10}+r\langle P\rangle_{10} &(10)\\
\frac{d}{dt}\langle m\rangle_{01} &= N_{\rm TF}k_{\rm on,TF}\langle m\rangle_{00}-(k_{\rm off,TF}+N_{\rm p}k_{\rm on,P}+\gamma)\langle m\rangle_{01}+(\beta^{-1}k_{\rm off,P}+\alpha r)\langle m\rangle_{11}+\alpha r\langle P\rangle_{11}, &(11)\\
\frac{d}{dt}\langle m\rangle_{10} &= N_{\rm p}k_{\rm on,P}\langle m\rangle_{00}-(N_{\rm TF}k_{\rm on,TF}+k_{\rm off,P}+r+\gamma)\langle m\rangle_{10}+\beta^{-1}k_{\rm off,TF}\langle m\rangle_{11} &(12)\\
\frac{d}{dt}\langle m\rangle_{11} &= N_{\rm p}k_{\rm on,P}\langle m\rangle_{01}+N_{\rm TF}k_{\rm on,TF}\langle m\rangle_{10}-(\beta^{-1}k_{\rm off,P}+\beta^{-1}k_{\rm off,TF}+\alpha r+\gamma)\langle m\rangle_{11}. &(13)
\end{aligned}$$

We obtain the equation for time evolution of the mean protein number ($\langle m\rangle$) by adding Eqns. A(10-13)

$$\begin{aligned}
\frac{d}{dt}\langle m\rangle &= \frac{d}{dt}\Big(\langle m\rangle_{00}+\langle m\rangle_{01}+\langle m\rangle_{10}+\langle m\rangle_{11}\Big),\\
&= r\langle P\rangle_{10}+\alpha r\langle P\rangle_{11}-\gamma\langle m\rangle &(14)
\end{aligned}$$

The steady state solutions can be obtained by setting the right hand side of the equations to zero which gives

$$\begin{aligned}
\langle m\rangle &= \frac{r}{\gamma}\Big(\langle P\rangle_{10}+\alpha\langle P\rangle_{11}\Big) &(15)\\
\langle P\rangle_{01} &= \frac{R}{1+R}-\frac{1+\beta R}{\beta(1+R)}\langle P\rangle_{11} &(16)\\
\langle P\rangle_{10} &= \frac{P}{1+P+V_2}-\frac{1+\beta(\alpha V_2+P)}{\beta(1+P+V_2)}\langle P\rangle_{11} &(17)\\
\langle P\rangle_{11} &= \frac{RP\beta\big(1+P+V_2+V_1+V_1R\big)}{P(1+R\beta)(1+P+V_2)+RV_1(1+R)(1+\alpha\beta V_2+P\beta)+(1+V_1+\alpha\beta V_2)(1+R)(1+P+V_2)} &(18)\\
\langle P\rangle_{00} &= 1-\langle P\rangle_{01}-\langle P\rangle_{10}-\langle P\rangle_{11} &(19)\\
\mathrm{FC} &= 1+\frac{\alpha\beta(1+P)-1-P\beta}{P\beta}\langle P\rangle_{11} &(20)\\
\mathrm{FC}_{\rm max} &= 1+\frac{\alpha\beta(1+P)-1-P\beta}{P\beta}\langle P\rangle_{11}(R\to\infty) &(21)\\
&= \frac{\alpha\beta(1+P+V_2)}{1+P\beta+\alpha\beta V_2}, &(22)
\end{aligned}$$

In the above equations we have substituted $R=N_{\rm TF}k_{\rm on,TF}/k_{\rm off,TF}$, $P=N_{\rm P}k_{\rm on,P}/k_{\rm off,P}$, $V_1=k_{\rm off,TF}/k_{\rm off,P}$, $V_2=r/k_{\rm off,P}$. Since $\langle P\rangle_{11}\geq 0$, for any concentration of TF, the nature of regulation (activation or repression) is determined by the expression $\alpha\beta(1+P)-1-P\beta$. When this quantity is greater(less) than one we obtain activation(repression). Also the condition for fold-change of 1 is independent of TF concentration and is given by the equation

$$\alpha=\frac{1+P\beta}{\beta(1+P)}. \tag{23}$$

The polymerase unbinding rate we use is $k_{\rm off,P}=1\ \mathrm{s}^{-1}$. The net polymerase binding rate ($N_{\rm P}k_{\rm on,P}$) is then determined from the promoter strength $P=N_{\rm P}k_{\rm on,P}/k_{\rm off,P}$ which is varied in the range $0.01-1$, the weak promoter

limit being 0.01 and 1 the strong promoter limit. Note that we do not tune the polymerase number independently. For the TF unbinding rate we use $k_{\mathrm{off,TF}} = 0.001\mathrm{s}^{-1}$ corresponding to LacI binding site O1 in *E. coli* [1]. We condense the TF number (N_{TF})and the sinlge TF binding rate ($k_{\mathrm{on,TF}}$) into one parameter which is varied in the range $0.0001-1$. We use cell-division time (τ) of 40 minutes corresponding to $\gamma = ln(2)/\tau = 0.0003$ s^{-1}.

Response time

To compute the response time we solve the ordinary differential equations (1-4 and 14) in MATLAB using the solver ode45. We assume that at time $t = 0$ the gene is expressing constitutively which gives the steady state expression level and the occupancies at $t = 0$ as

$$\langle m_{t=0} \rangle = \frac{rP}{\gamma(1 + P + V_2)}, \tag{24}$$

$$\langle P_{t=0} \rangle_{00} = \frac{1 + V_2}{1 + P + V_2}, \tag{25}$$

$$\langle P_{t=0} \rangle_{01} = 0, \tag{26}$$

$$\langle P_{t=0} \rangle_{10} = \frac{P}{1 + P + V_2}, \tag{27}$$

$$\langle P_{t=0} \rangle_{11} = 0. \tag{28}$$

At time ($t = 0$), TFs are instantly switched to an active state. Once actively regulated by TFs, the expression level will change before eventually reaching a new steady state given by Eqns A(15-19). The response time is then computed as the time for the expression to reach halfway from the prior, unregulated state level to the new regulated level.

II. DERIVATION OF FOLD-CHANGE FROM THERMODYNAMIC MODEL

Here we derive the expression for fold-change (Eqn 1 and Fig 1 in main text) using thermodynamic model, which can be also be be found in [2]. We assume that $N_{\rm P}$ number of RNAPs and $N_{\rm TF}$ number TFs are distributed in the genome with $N_{\rm ns}$ nos-specific binding sites (the size of the genome) and one binding site for RNAP and one for TFs in the promoter. In order to find the probability of a particular state, we need to find the number of ways the state can be realized multiplied by its Boltzmann weight ($\exp(-\epsilon/k_{\rm B}T)$). For example, the state where both the binding sites are empty, this can be written as

$$Z_{\rm Free} \;=\; \frac{N_{\rm ns}!}{N_{\rm TF}!N_{\rm P}!(N_{\rm ns}-N_{\rm P}-N_{\rm TF})!}e^{-(N_{\rm P}\epsilon_{\rm P,ns}+N_{\rm TF}\epsilon_{\rm TF,ns})/k_{\rm B}T}. \tag{29}$$

Similarly, the other sates can be written as

$$Z_{\rm Pol-bound} \;=\; \frac{N_{\rm ns}!}{N_{\rm TF}!(N_{\rm P}-1)!(N_{\rm ns}-N_{\rm P}+1-N_{\rm TF})!}e^{-(\epsilon_P+(N_{\rm P}-1)\epsilon_{\rm P,ns}+N_{\rm TF}\epsilon_{\rm TF,ns})/k_{\rm B}T}, \tag{30}$$

$$Z_{\rm TF-bound} \;=\; \frac{N_{\rm ns}!}{(N_{\rm TF}-1)!N_{\rm P}!(N_{\rm ns}-N_{\rm P}-N_{\rm TF}+1)!}e^{-(\epsilon_{TF}+N_{\rm P}\epsilon_{\rm P,ns}+(N_{\rm TF}-1)\epsilon_{\rm TF,ns})/k_{\rm B}T}, \tag{31}$$

$$Z_{\rm Co-bound} \;=\; \frac{N_{\rm ns}!}{(N_{\rm TF}-1)!(N_{\rm P}-1)!(N_{\rm ns}-N_{\rm P}-N_{\rm TF}+2)!}e^{-(\epsilon_P+\epsilon_{TF}+\epsilon_I+(N_{\rm P}-1)\epsilon_{\rm P,ns}+(N_{\rm TF}-1)\epsilon_{\rm TF,ns})/k_{\rm B}T}. \tag{32}$$

Each term above can be re-scaled by dividing with $Z_{\rm Free}$ to obtain the likelihood shown in Fig 1C. The partition function ($Z_{\rm Total}$) then can be expressed as the sum of the individual weights and the probability of being in a state is then given by

$$P_{\rm Free}=\frac{Z_{\rm Free}}{Z_{\rm Total}},\quad P_{\rm Pol-bound}=\frac{Z_{\rm Pol-bound}}{Z_{\rm Total}},\quad P_{\rm TF-bound}=\frac{Z_{\rm TF-bound}}{Z_{\rm Total}},\quad P_{\rm Co-bound}=\frac{Z_{\rm Co-bound}}{Z_{\rm Total}}. \tag{33}$$

The gene expression then can be found by adding the probability of Pol-bound and Co-bound state multiplied by the rate of production in those states, and since pol-bound and co-bound states are the only productive states,the net gene-expression is $m = rP_{\rm Pol-bound} + \alpha r P_{\rm co-bound}$. Here, α is the acceleration term which modifies the transcription rate in co-bound state.The fold-change of gene-expression is after doing some algebra is

$$\begin{aligned}
{\rm Fold-change}| \;&=\; \frac{\rm Expression\,in\,presence\,of\,TF}{\rm Expression\,without\,TF},\\
&=\; \frac{1+\alpha\frac{N_{\rm TF}}{N_{\rm ns}}e^{-(\Delta\epsilon_{TF}+\Delta\epsilon_I)/k_BT}}{1+\frac{N_{TF}}{N_{ns}}e^{-\Delta\epsilon_{TF}/k_BT}\frac{1+\frac{N_P}{N_{ns}}e^{-(\Delta\epsilon_P+\Delta\epsilon_I)/k_BT}}{1+\frac{N_P}{N_{ns}}e^{-\Delta\epsilon_P/k_BT}}},\\
&=\; \frac{1+\alpha\beta R}{1+R\frac{1+P\beta}{1+P}}.
\end{aligned} \tag{34}$$

In the above equation we have used $\Delta\epsilon_P = \epsilon_P - \epsilon_{P,ns}$, $\Delta\epsilon_{TF} = \epsilon_{TF} - \epsilon_{TF,ns}$, $\beta = \exp(-\Delta\epsilon_I/k_BT)$, $R = \exp(-\Delta\epsilon_{TF}/k_BT)N_{TF}/N_{ns} = N_{\rm TF}/k_D^{\rm TF}$, and $P = \exp(-\Delta\epsilon_P/k_BT)N_P/N_{ns} = N_{\rm P}/k_D^{\rm P}$. The equation above can further be re-arranged to,

$${\rm Fold-change} = \frac{1+\chi{\rm FC_{max}}}{1+\chi}, {\rm with}\ {\rm FC_{max}} \;=\; \frac{\alpha\beta(1+P)}{(1+\beta P)}\ {\rm and}\ \chi = \frac{(1+\beta P)}{(1+P)}R. \tag{35}$$

III. FITTING MODEL TO PRE-EXISTING DATA

In Fig 2F, we show two examples of data fit to our model. For the LacI repression data from Lanzer and Bujard [3] we chose data points with the following constraints: (1) The LacI binding site was in the natural, proximal position, corresponding to O3 constructs in their nomenclature, (2) the promoter is not regulated by other TFs (such as CRP) and (3) the availability of direct promoter strength measurements in related work from Brunner and Bujard [4]. This leaves 5 data points corresponding to promoters Plac.L8, Ptac, Pcon/03, PA1/03, PN25/03. We then plot the fold-change (inverse of the reported repression factor) as a function of promoter strength (which is linearly related to the reported K_{eq} times a fit parameter representing the effective RNAP concentration). Error bars represent error propagation method from their reported uncertainty in repression and measured uncertainty in k_{on}. The relationship $FC = \alpha\beta(1+P)/(1+\beta P)$ was then fit to the data.

For the regulation by AraC (right panel in Fig 2F). We estimated the background fluorescence by extrapolating the signal in their expression data as promoter strength goes to 0. We subtracted this number from all expression data and calculated mean fold-change from repeated measurements of each promoter. We report error bars on each data point resulting from the propagation of errors of the uncertainty in each fluorescence measurement and the uncertainty in the background value. Promoter strength, P was calculated using the relationship to the constitutive expression of each promoter, c: $P = 1/((r/c) - 1)$, where r represents the constitutive expression of the strongest promoter (when $P \to \infty$). We then fit $FC = \alpha\beta(1+P)/(1+\beta P)$ with α, β and r as fit parameters. We exclude low expression data (red shaded area) with large uncertainty compared to the background value (when $P \to 0$).

IV. NOISE IN GENE EXPRESSION

We compute coefficient of variation (CV) as a measure of noise. In order to do that we obtain the second moments by multiplying the Eqns. (1-4) by m^2 and summing over $m = 0$ to $m = \infty$ given by

$$\begin{aligned}
\frac{d}{dt}\langle m^2\rangle_{00} &= -(N_{\rm TF}k_{\rm on,TF} + N_{\rm P}k_{\rm on,P})\langle m^2\rangle_{00} + k_{\rm off,TF}\langle m^2\rangle_{01} + k_{\rm off,P}\langle m^2\rangle_{10} + r\langle (m+1)^2\rangle_{10} \\
&\quad -\gamma\langle 2m^2 - m\rangle_{00}, \qquad (36)\\
\frac{d}{dt}\langle m^2\rangle_{01} &= N_{\rm TF}k_{\rm on,TF}\langle m^2\rangle_{00} - (k_{\rm off,TF} + N_{\rm P}k_{\rm on,P})\langle m^2\rangle_{01} + \beta^{-1}k_{\rm off,P}\langle m^2\rangle_{11} + \alpha r\langle (m+1)^2\rangle_{11} \\
&\quad -\gamma\langle 2m^2 - m\rangle_{01}, \qquad (37)\\
\frac{d}{dt}\langle m^2\rangle_{10} &= N_{\rm P}k_{\rm on,P}\langle m^2\rangle_{00} - (k_{\rm off,P} + N_{\rm TF}k_{\rm on,TF})\langle m^2\rangle_{10} + \beta^{-1}k_{\rm off,TF}\langle m^2\rangle_{11} - r\langle m^2\rangle_{10} - \gamma\langle 2m^2 - m\rangle_{10}, \qquad (38)\\
\frac{d}{dt}\langle m^2\rangle_{11} &= N_{\rm P}k_{\rm on,P}\langle m^2\rangle_{01} + N_{\rm TF}k_{\rm on,TF}\langle m^2\rangle_{10} - \beta^{-1}(k_{\rm off,P} + k_{\rm off,TF})\langle m^2\rangle_{11} - \alpha r\langle m^2\rangle_{11} - \gamma\langle 2m^2 - m\rangle_{11}, \qquad (39)\\
\frac{d}{dt}\langle m^2\rangle &= \frac{d}{dt}(\langle m^2\rangle_{00} + \langle m^2\rangle_{01} + \langle m^2\rangle_{10} + \langle m^2\rangle_{11}), \qquad (40)\\
&= r\langle 2m+1\rangle_{10} + \alpha r\langle 2m+1\rangle_{11} - \gamma\langle 2m^2 - m\rangle. \qquad (41)
\end{aligned}$$

The steady state second moments are obtained by setting the above equations to zero which along with the steady states first moment gives

$$\langle m^2\rangle = \langle m\rangle + \frac{r}{\gamma}\big(\langle m\rangle_{10} + \alpha\langle m\rangle_{11}\big). \qquad (42)$$

In order to compute CV or Fano factor we first numerically estimate the moments $\langle m\rangle_{10}$ and $\langle m\rangle_{11}$ and then use the the following equations,

$$\begin{aligned}
{\rm CV}^2 &= \frac{\langle m^2\rangle - \langle m\rangle^2}{\langle m\rangle^2}, \qquad (43)\\
{\rm Fano} &= \frac{\langle m^2\rangle - \langle m\rangle^2}{\langle m\rangle}. \qquad (44)
\end{aligned}$$

V. AUTOREGULATING GENE

The set of ordinary differential equations governing the dynamics of an auto-regulating gene can be written as

$$\frac{dP_{00}}{dt} = -(mk_{\mathrm{on,TF}} + N_{\mathrm{P}}k_{\mathrm{on,P}})P_{00} + (k_{\mathrm{off,TF}} + \gamma)P_{01} + (k_{\mathrm{off,P}} + r)P_{10}, \tag{45}$$

$$\frac{dP_{01}}{dt} = mk_{\mathrm{on,TF}}P_{00} - (k_{\mathrm{off,TF}} + N_{\mathrm{P}}k_{\mathrm{on,P}} + \gamma)P_{01} + (\beta^{-1}k_{\mathrm{off,P}} + \alpha r)P_{11}, \tag{46}$$

$$\frac{dP_{10}}{dt} = N_{\mathrm{P}}k_{\mathrm{on,P}}P_{00} - (k_{\mathrm{off,P}} + mk_{\mathrm{on,TF}})P_{10} + (\beta^{-1}k_{\mathrm{off,TF}} + \gamma)P_{11} - rP_{10}, \tag{47}$$

$$\frac{dP_{11}}{dt} = N_{\mathrm{P}}k_{\mathrm{on,P}}P_{01} + mk_{\mathrm{on,TF}}P_{10} - \big(\beta^{-1}(k_{\mathrm{off,P}} + k_{\mathrm{off,TF}}) + \alpha r + \gamma\big)P_{11}, \tag{48}$$

$$\frac{dm}{dt} = rP_{10} + \alpha rP_{11} - \gamma m - mk_{\mathrm{on,TF}}(P_{00} + P_{10}) + k_{\mathrm{off,TF}}(\beta^{-1}P_{11} + P_{01}). \tag{49}$$

We numerically evaluate the steady state expression level by setting the above equations to zero. To compute the response time we solve the ordinary differential equation using ode45 in MATLAB.

We further looked into how the auto-regulated genes behave if the TF is a dimer. We modified the Eqns. E1-E6, and added an extra step where the TFs now act as a dimer. The monomers (m) becomes a dimer (m_d) with a rate k_{dim} and again can dissociate to form monomer at a rate k_{mon}. The set of ODEs governing this can be written as,

$$\frac{dP_{00}}{dt} = -(m_dk_{\mathrm{on,TF}} + N_{\mathrm{P}}k_{\mathrm{on,P}})P_{00} + (k_{\mathrm{off,TF}} + \gamma)P_{01} + (k_{\mathrm{off,P}} + r)P_{10}, \tag{50}$$

$$\frac{dP_{01}}{dt} = m_dk_{\mathrm{on,TF}}P_{00} - (k_{\mathrm{off,TF}} + N_{\mathrm{P}}k_{\mathrm{on,P}} + \gamma)P_{01} + (\beta^{-1}k_{\mathrm{off,P}} + \alpha r)P_{11}, \tag{51}$$

$$\frac{dP_{10}}{dt} = N_{\mathrm{P}}k_{\mathrm{on,P}}P_{00} - (k_{\mathrm{off,P}} + m_dk_{\mathrm{on,TF}})P_{10} + (\beta^{-1}k_{\mathrm{off,TF}} + \gamma)P_{11} - rP_{10}, \tag{52}$$

$$\frac{dP_{11}}{dt} = N_{\mathrm{P}}k_{\mathrm{on,P}}P_{01} + m_dk_{\mathrm{on,TF}}P_{10} - \big(\beta^{-1}(k_{\mathrm{off,P}} + k_{\mathrm{off,TF}}) + \alpha r + \gamma\big)P_{11}, \tag{53}$$

$$\frac{dm}{dt} = rP_{10} + \alpha rP_{11} + 2k_{\mathrm{mon}}m_d - 2k_{\mathrm{dim}}m^2 - \gamma m, \tag{54}$$

$$\frac{dm_d}{dt} = -k_{\mathrm{mon}}m_d + k_{\mathrm{dim}}m^2 - m_dk_{\mathrm{on,TF}}(P_{00} + P_{10}) + k_{\mathrm{off,TF}}(\beta^{-1}P_{11} + P_{01}) - \gamma m_d. \tag{55}$$

Overall, the qualitative remains intact when the TF acts as a dimer. However, depending on how fast the monomers becomes a dimer or the dimer dissociates to form monomer, the response times vary significantly (see Fig. 1).

[1] M. Z. Ali, V. Parisutham, S. Choubey, and R. C. Brewster, Inherent regulatory asymmetry emanating from network architecture in a prevalent autoregulatory motif, Elife **9** (2020).

[2] S. Guharajan, S. Chhabra, V. Parisutham, and R. C. Brewster, Quantifying the regulatory role of individual transcription factors in *escherichia coli*, Cell Reports **37**, 109952 (2021).

[3] M. Lanzer and H. Bujard, Promoters largely determine the efficiency of repressor action, Proc Natl Acad Sci U S A **85**, 8973 (1988).

[4] M. Brunner and H. Bujard, Promoter recognition and promoter strength in the *Escherichia coli* system, Embo J **6**, 3139 (1987).

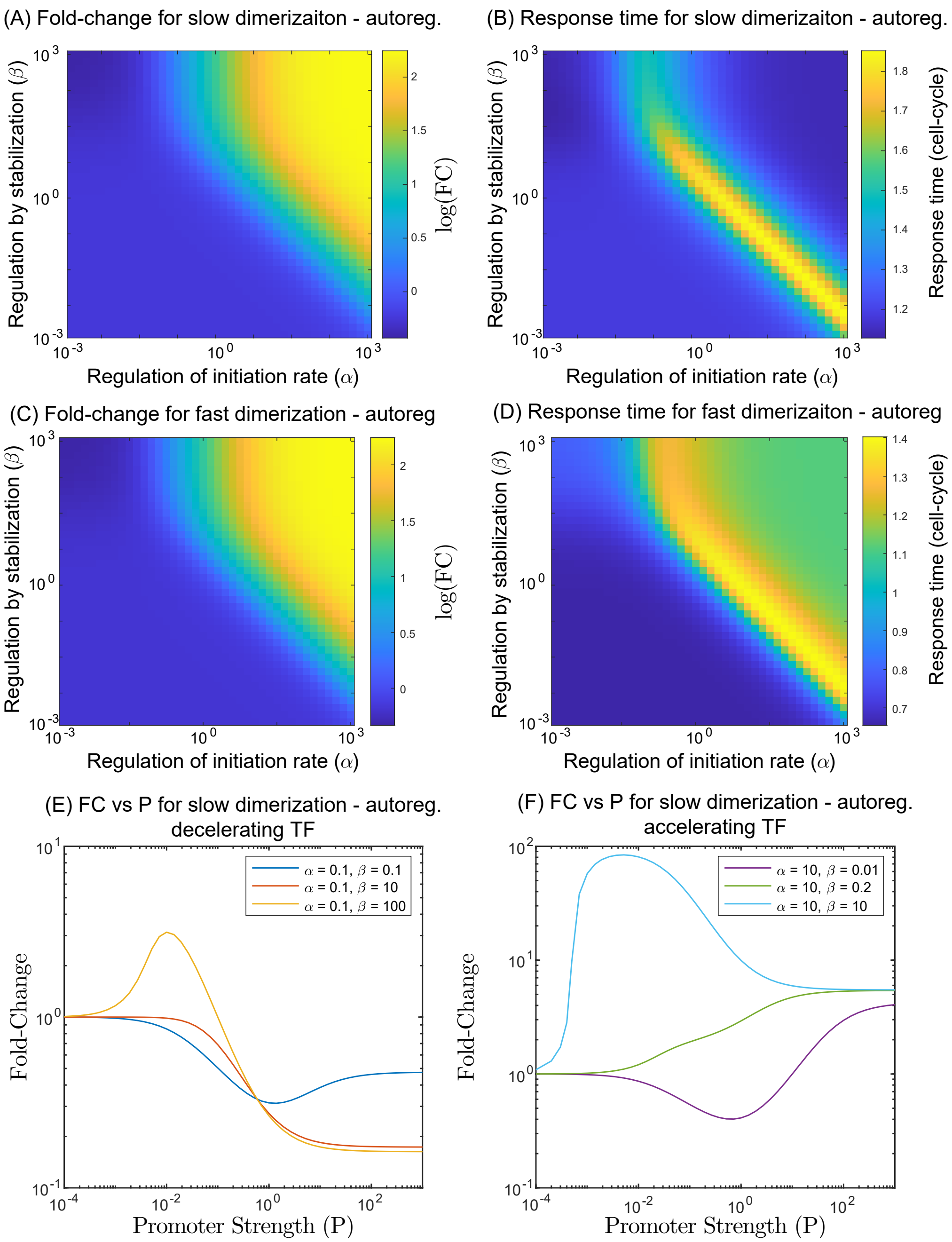

FIG. 1. **Behavior of auto-regulated genes when TFs act as a dimer.** Fold-change (A,C) and response time (B,D) of autoregulating gene for slow dimerization ($k_{\mathrm{dim}} = 0.1\mathrm{s}^{-1}\mathrm{TF}^{-1}$, $k_{\mathrm{mon}} = 1\mathrm{s}^{-1}$, Panel A,B) and fast dimerization ($k_{\mathrm{dim}} = 1\mathrm{s}^{-1}\mathrm{TF}^{-1}$, $k_{\mathrm{mon}} = 0.1\mathrm{s}^{-1}$, Panel C,D). (E,F) The fold-change of auto-regulating gene versus titration of promoter strength. The qualitative feature of TF switching from activator to repressor when $\alpha < 1$ and $\alpha\beta > 1$ (yellow curve in E) as well as switching from repressor to activator when $\alpha > 1$ and $\alpha\beta < 1$ is preserved (magenta curve in F).
