# Supplementary figures and images for: Regulatory properties of transcription factors with diverse mechanistic function
Source: PLoS Comput Biol. 2024 Jun 10;20(6):e1012194. doi: 10.1371/journal.pcbi.1012194 (PMC11192337; doi:10.1371/journal.pcbi.1012194)

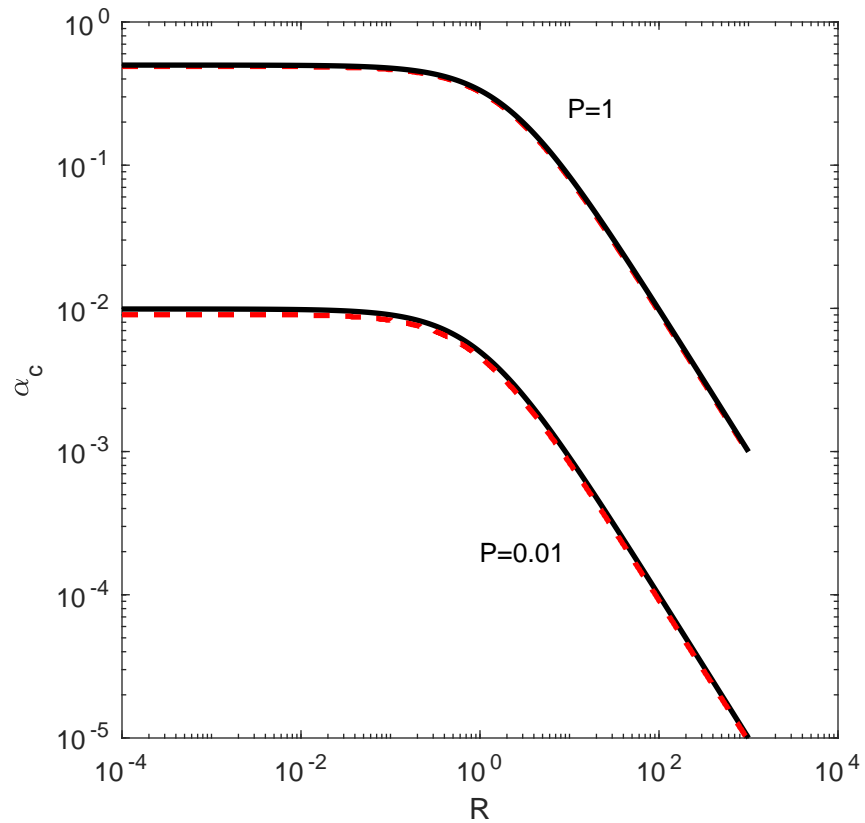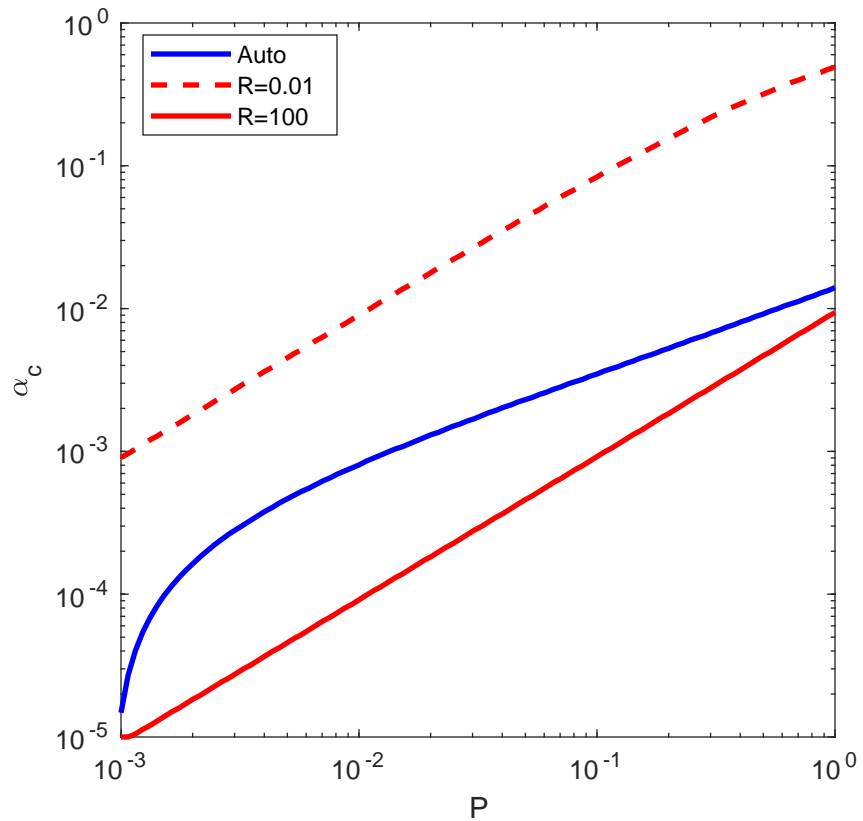

Supplement: S1 Fig — (A) αc from the thermodynamic model (black lines) and the full model (red lines) as a function of TF concentration for weak (P = 0.01) and strong promoter (P = 1). In order to find αc for the full model, we compute fold-change as a function of β for a range of α values with small increments. We then find the value of alpha where the fold-change vs β curve switches from monotonically decreasing to monotonically increasing. (B) αc for autoregulated gene (blue line) and the genes regulated by fixed TF concentration (red lines) as a function of promoter strength. (PDF) [file pcbi.1012194.s002.pdf]

(A) Response time vs Fold-change

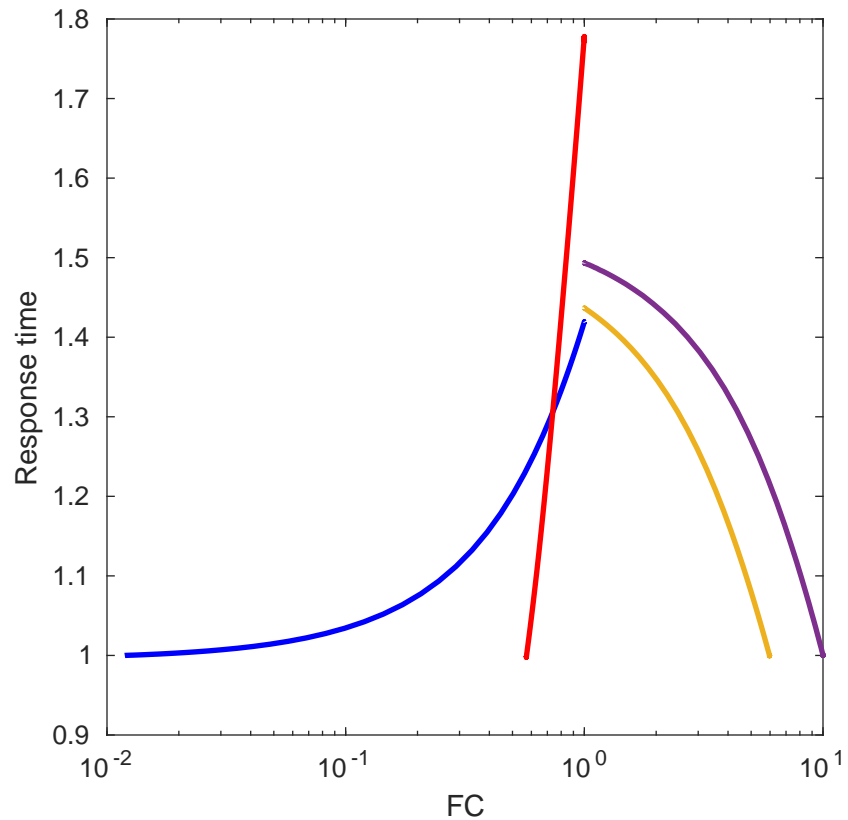

(B) Response time vs TF concentration

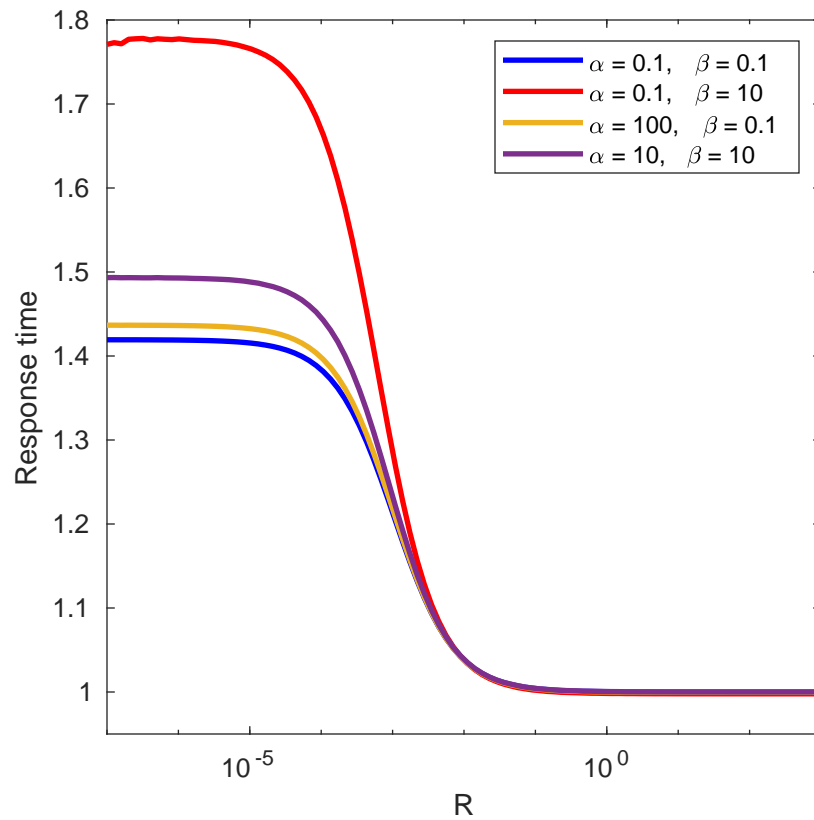

Supplement: S2 Fig — Response time as a function of fold-change (A) or TF concentration (B) as the TF concentration is tuned. As the TF concentration approaches to infinity the response time is saturated to one cell-cycle irrespective of the regulatory parameter of the TF. (PDF) [file pcbi.1012194.s003.pdf]
