# Supplementary material for: Regulatory properties of transcription factors with diverse mechanistic function
Source: PLoS Comput Biol. 2024 Jun 10;20(6):e1012194. doi: 10.1371/journal.pcbi.1012194 (PMC11192337; doi:10.1371/journal.pcbi.1012194)
Supplement: S3 Fig — Fold-change (A,C) and response time (B,D) of autoregulating gene for slow dimerization (kdim = 0.1s−1TF−1, kmon = 1s−1, Panel A,B) and fast dimerization (kdim = 1s−1TF−1, kmon = 0.1s−1, Panel C,D). (E,F) The fold-change of auto-regulating gene versus titration of promoter strength. The qualitative feature of TF switching from activator to repressor when α < 1 and αβ > 1 (yellow curve in E) as well as switching from repressor to activator when α > 1 and αβ < 1 is preserved (magenta curve in F). (PDF) [file pcbi.1012194.s004.pdf]

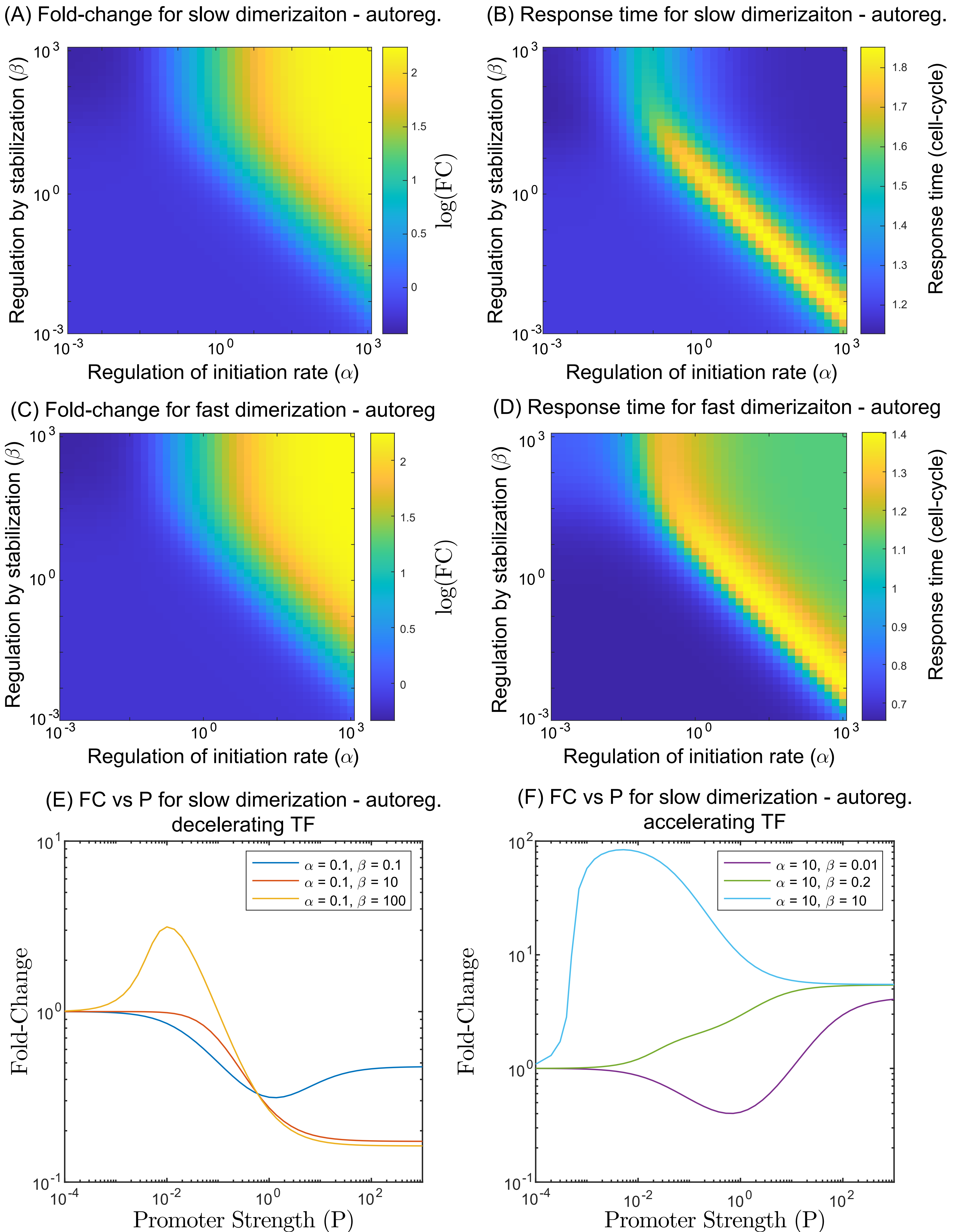
(A) Fold-change for slow dimerizaiton - autoreg.
Regulation by stabilization (β)
Regulation of initiation rate (α)
log(FC)
(B) Response time for slow dimerizaiton - autoreg.
Regulation by stabilization (β)
Regulation of initiation rate (α)
Response time (cell-cycle)
(C) Fold-change for fast dimerization - autoreg
Regulation by stabilization (β)
Regulation of initiation rate (α)
log(FC)
(D) Response time for fast dimerizaiton - autoreg
Regulation by stabilization (β)
Regulation of initiation rate (α)
Response time (cell-cycle)
(E) FC vs P for slow dimerization - autoreg.
decelerating TF
α = 0.1, β = 0.1
α = 0.1, β = 10
α = 0.1, β = 100
Fold-Change
Promoter Strength (P)
(F) FC vs P for slow dimerization - autoreg.
accelerating TF
α = 10, β = 0.01
α = 10, β = 0.2
α = 10, β = 10
Fold-Change
Promoter Strength (P)
