# Supplementary material for: Regulatory properties of transcription factors with diverse mechanistic function
Source: PLoS Comput Biol. 2024 Jun 10;20(6):e1012194. doi: 10.1371/journal.pcbi.1012194 (PMC11192337; doi:10.1371/journal.pcbi.1012194)
Supplement: Attachment — Submitted filename: EditorLetter.pdf [file pcbi.1012194.s005.pdf]

Dear Editors Klumpp and Mendes,

We thank the reviewers and editors for the thoughtful assessment of our manuscript. We were very pleased with the positive nature of the three referee responses. In response to the concerns raised by each referee, we have modified the original submission to address these points. All changes are highlighted in red in the revised manuscript. Please find below our responses to each specific critique in addition to information on how we addressed the critique. We believe that these changes have improved the manuscript and make it an excellent candidate for publication in PLoS Comp Bio.

On a personal note, we have a grant review in early June which would benefit greatly from acceptance of this manuscript. Although the reviewer comments were extensive we agree with your assessment that “all reviewers were basically positive” and I hope it is clear we have taken the comments seriously and put significant work into answering each in a point-by-point fashion. As such, I would like to request that if an acceptance letter could be generated as soon as feasible (even if we still require additional revisions) it would be very appreciate

Thank you,

Md Zulfikar Ali

Sunil Guharajan

Vinuselvi Parisutham

Robert Brewster

Reviewer 1:

In the manuscript, the authors describe their model as using the simplest regulatory architecture, meaning that the promoter is regulated only by a single TF and that the TF has only one binding site on the promoter. It is said, that modeling approaches are a focus in the field of regulatory biology. Are there others/similar models available in the literature and what are the pros/cons and limitations compared to the model presented?

There are many different models of gene regulation. Probably the two most popular types are the two mentioned here: kinetic and thermodynamic. Within those broad classifications, models range from phenomenological to highly detailed. As such, the ideas that compose the foundation of our model are not unique and we hope that we have been fair to those other works in our introduction. However, we believe that writing this model as a general way to think about TF function is an important idea; we highlight that “activator” or “repressor” isn’t an intrinsic property of a TF but rather is the outcome of a rather complicated relationship between the modes of regulation of a TF and the promoter it is acting on. Given this, we then extend this idea to look at the implied characteristics of TFs with wide ranges in these regulatory characteristics such as noise and timing.

Is it possible to apply the model to literature data as it was done before in their previous work for Escherichia coli to verify the results?

We now include a comparison of our theory predictions to data from Lanzer and Bujard (PNAS 1988, PMC282639) for simple regulation by LacI or the 4 promoters where the Keq is measured (Brunner and Bujard 1987). LacI is interesting because it's one of few model TFs where we know that it operates (in the natural, proximal binding location) through \beta < < 1 (for example from Sanchez, *et al.* Embo J 2011 PMC3209775). We find this data is well fit by our model both qualitatively (stronger promoters are less repressed) and quantitatively. However, our model offers an alternative explanation for their data; The authors explanation is that strong promoters can bind polymerase "faster" which makes them harder to repress because there is a kinetic competition of on-rates that would not be captured in an equilibrium model. Here we are pointing out that an equilibrium model actually predicts exactly this relationship between TFs which are destabilizing (as we know LacI is) and their promoters.

We also show the model against the data from Chen et al (PMC5754348) for the TF AraC. This is a nice data set because the mechanisms of AraC are studied and known to fall under "beta>1" in our model (Zhang, Reeder and Schleif 1996 JMB PMID: 8613984). It should be noted that the authors already do fit a stabilizing-only model to their data in their work, we make note of this in the text. Overall our goal is to show that our model can fit diverse data sets for TFs using data where the mechanisms are known to validate the concept.

We have added this figure panel to Fig 2 and added a new section in the results titled "Applications of general model to data".

When introducing the fold-change in Equation 1, you reference to [39]. In [39] Fig. 1A you use a different definition for FCmax. Could you explain where the difference in the model comes from?

Thank you for pointing this out. The difference arises from the "weak promoter assumption" we made in that paper (i.e. that 1+P ≅ 1). This is an assumption we had used in our research for that specific promoter for years (for example, Garcia and Phillips PNAS 2011, Brewster et al Cell 2014, Jones Brewster and Phillips Science 2014). As we began to consider diverse promoters, it was clear that this was no longer a justifiable assumption and so the equation in Fig 1A is the full expression. We now explicitly point this out after introducing the predictions of Fig. 1C.

Minor concerns:

In Fig. 1B, it would be nice to indicate the role of β by highlighting the altered rate.

Thank you for catching this oversight, as written beta is not in the model! We have added beta into the appropriate rate here.

"The TF and the polymerase unbind/dissociate from the bound states at the rate that is independent of the TF/polymerase concentration and is only dependent on the interaction between the TF and the polymerase, and binding site identity. We denote the unbinding rates of the TF and polymerase as kTF and kP, respectively."
In Fig. 1B: If the unbinding rate is dependent on the interaction between the TF and the polymerase, why are kTF and kP the same for the transitions from either TF bound or polymerase bound to the empty state and the TF and polymerase co-bound state to the other states? Why is the unbinding rate not different coming from the co-bound state?

Exactly correct, this is a mistake in Fig 1B. Thank you for pointing it out! We have now fixed it.

On page 3 you reference (Fig. 2B, right panel). Is this supposed to be Fig. 2C?

That is correct, thank you for pointing out this mistake. We have fixed it in the manuscript.

On page 4: "Here, V2 = r/kP is the ratio of the transcription rate from polymerase bound state and the polymerase unbinding rate without TF (kP,off)."
In the equation for V2, is it supposed to be kP,off or kP?

Again this is correct, this has been fixed in the manuscript. To be consistent, now we use k_{off, P} for polymerase off-rate and k_{off,TF} for TF- off rate throughout the manuscript.

Page 5: "A heat map showing the response time in α − β space is shown in Fig. 4B. We find that for strong stabilization and deceleration (α < 1) the response times are the longest and reach several cell-cycles." Just for clarity it would be nice to add (β > 1) after strong stabilization.

We have added this suggestion for clarity.

"Code and results are available on Github." - I could not find a link anywhere.

We have added a link to the github repository here: https://github.com/zulfikgp/TFFunction

Reviewer 2:
The manuscript is well-written and easy to follow, with instructive figures that help with illustration of the main points. The reviewer has few minor points that can help with bridging the gap between the simplifying assumptions made in the model and the reality of gene regulation (even in prokaryotes).
Comments
1. The model, as presented, holds for prokaryotes. It would be great if the authors include few points of discussion about what modifications of the model, at minimum, would be needed to render it applicable to study gene regulation in eukaryotic systems.

We have now added some points about Eukaryotic transcription in the discussion. Importantly, similar models (although in a kinetic framework) have been used to describe Eukaryotic transcription (See for example, Martinez-Corral et al, Cell Systems 2023 PMC10472254 and Scholes et al, Cell Systems 2017 PMC5469051). There will be advantages to that type of model (generality) and disadvantages (interpretability and complexity, number of parameters, etc).

The papers above focused more on the interplay between different TFs and their specialized roles in regulation, rather than the relationship with the promoter but the ideas will still hold. Further, recent studies have shown interesting relationships between regulators and promoters predicted here in measurements of individual effector domains acting on different promoters (Mukund et al Cell Sys 2023), our model framework would predict these relationships even if the detailed mechanisms are more complex. We now elaborate these points in the discussion.

2. Could the authors comment on the effects of having a promoter regulated by more than one transcription factor? To what extent could any of the conclusions from the simple model hold? This is relevant for establishing the relation between model and actual regulation in prokaryotic systems.

This is an extremely important point. One of the advantages to understanding what individual TFs do when they regulate a gene is to gain insight into what we should expect when they act in combination. This has been tried through the lens of “activators” and “repressors”, but what we are highlighting here is that this is not the bottom-level of information, in fact it’s insufficient even to predict what a single TF does on different promoters. From this model it is relatively trivial to extend to two TFs (the model goes from 4 states to 7), the predictions will be predicated on parameters such as the regulatory interactions of each TF individually (which can be measured through our model) and the net interactions for the 2-TF bound state, which will be the one new parameter. The crucial point is that our approach gives us a foot-in-the-door, we have some information about what to expect in the first place, which we have never had before. Our approach allows for interpretation of multi-TF regulation with a set of informed hypothesis (i.e., maybe the individual effects add, maybe they multiply, maybe the dominant one wins etc) that can be precisely tested.

3. Could authors comment how the numerical estimation of the parameter alpha_c. This was not explained in the methods or the main part of the text.

We now explain this estimation in the Fig. S1 caption. In order to find α_c for the full model, we compute fold-change as a function of β for a range of α values with small increments. We then find the value of alpha where the fold-change vs β curve switches from monotonically decreasing to monotonically increasing.

4. Fig. 1, spell out Txn rate on panel C.

Added the full spelling for transcription.

Reviewer 3:

- This manuscript needs a discussion on realistic ranges of parameters for transcription factors found in nature. The example values are often orders of magnitude apart, so it might be helpful to discuss whether these parameters could plausibly have such a wide range. Ideally, the authors should find examples where stabilization and acceleration can be calculated from existing data. Equilibrium constants of the TF-RNAP-DNA complex or transcription initiation rates might not be readily available, but at the minimum the authors should comment on possible means to measure stabilization and acceleration, as well as comment on what are reasonable ranges expected to be found in nature. Recent studies on the properties of the parameter space of transcriptional regulation did a similar analysis (PMID: 36168291).

Thank you for this suggestion. The difficulty in estimating “limits” of these parameters is that, as with any ratio, they can get arbitrarily large/small when one of the two factors gets very small. As such, there are no “hard” bounds to alpha and beta. This does highlight that in these extreme scenarios, a ratiometric approach isn’t the right choice (for instance if r is approximately 0 in the absence of TF). You can do back-of-the-envelope calculation for some limits such as the fastest initiation rate is limited by how fast a transcribing polymerase can clear the promoter (~1/s).

Primarily we have chosen numbers here for \alpha and \beta which conform to our expectations for fold-changes from promoters we have measured in previous (as well as unpublished) experiments on different TFs regulating diverse promoters. In those experiments we typically see maximum effects on the order of several hundred to a thousand. As such, we plot these mechanisms in the ranges that produce those fold-changes.

There is a bit of a resolution problem at the very low end. It is very difficult to measure the difference between 1000-fold repression and 100000-fold repression in most experiments and they are both synonymous with 0 in most cases.

However, that being said, it is definitely a significant interest of ours to measure these parameters using *in vitro* techniques. There are some measurements, for instance between LacI binding at the proximal binding position and polymerase (Sanchez, *et al.* Embo J 2011 PMC3209775). Here they show a near complete blockage of co-binding to their measurement precision. We have also added some data on this TF, supported by the mechanisms measured *in vitro* to the manuscript under the new section “Applications of general model to data” Finally, we now discuss the use of *in vitro* techniques to measure alpha and beta on promoters in the first paragraph of the conclusions.

- Although the model of gene regulation by a single TF yields interesting results, the manuscript does not address the effects of cooperativity, which is the norm in transcriptional regulation. The addition of such nonlinearities to the model is particularly relevant to the case of self-regulation, and could potentially reveal new properties. Implementation of dimerized binding of transcription factors within this framework is relatively straightforward, and, in my opinion, would significantly expand the scope of the results. But at least a discussion would be very useful.

It is true that cooperativity/dimerization is a common theme in regulatory biology. We focused on simple binding/unbinding kinetics of single TFs since the focus here was about characterizing TF function. The interplay between more complex models of TF binding and TF function should produce interesting phenomena. We have now added a section on the effect of dimerization of TF on autoregulatory properties in the supplement. Although the quantitative behavior is shifted, qualitatively the results are similar. We now highlight this in the Appendix E.

[We do not have a reference to dimerization in Main text]

- Some of the key findings of this manuscript are not obvious, and would greatly benefit from more intuitive explanations. The authors provide such explanation when showing why incoherent promoters can be activators or repressors depending on promoter strength. However, it is still not obvious to me why higher stabilization decreases fold-change at finite TF concentrations with low acceleration. Those conditions also seem to result in the longest response times. Similarly, there are other results throughout the paper that could be better interpreted.

We have gone through our results to add more of such intuitive explanations to some of the results. We have now added an intuitive explanation for the curious result of high stabilization and low acceleration producing lower FC values than low stabilization and low acceleration when TF number is limiting:
“This counter-intuitive result occurs because at finite TF concentrations, the promoter can express both with and without the TF bound. In these cases, the TF-RNAP cobound state becomes extremely stable ($\beta\gg1$) and transcriptionally unproductive ($\alpha \ll 1$; this specific combination of parameters locks the gene into a state of low activity. When the complex is less stable ($\beta<1$), it will allow for relatively increased expression from the RNAP-only bound state.”

We have also added an intuitive explanation in the timing section that mirrors this idea and relates it to the phenomenon in FIg. 3A.

Minor points:

- The derivation of the thermodynamic model should be better explained and discussed, perhaps a section in the supporting material would help. For instance, it is not immediately obvious to me why the effective concentration of the TF (chi) depends on beta. Presumably the complex formed with RNAP stabilizes the binding of the TF itself, as can be inferred from the panel in Fig 1C (it could be the case that TF binding does not depend on RNAP presence). However, this is not discussed in the text, and there is no reference to beta in the diagram in Fig 1B.

This is exactly right regarding the coupling between P and beta in chi. We have significantly expanded this discussion right below the equations introducing the idea in the model section. We have also added a section to the supplement that more thoroughly derives and describes the thermodynamic model.

- In the Introduction, the authors mention “Our model is primarily suited for prokaryotic gene regulation, however it can be generalized for eukaryotic systems as well.” What aspects of eukaryotic gene regulation would need to be added to their modeling framework?

One significant thing not included in our model, which is relevant for eukaryotic regulation, is genome accessibility. The other aspect of our formulation that may not be well-suited for eukaryotics is that we frame regulatory effects as fold-changes in the rates of things. For eukaryotic systems, the basal levels of RNAP binding or initiation are low without the intervention of TFs. That being said, similar ideas to those in our models have been applied to regulation of eukaryotic systems by several labs (see, for instance, Martinez-Corral et al, PMC10472254). This was discussed briefly in the introduction, but not in the context of prokaryotic vs eukaryotic. We now add references to similar modeling approaches used in Eukaryotic contexts into the first paragraph of the discussion.

- In Fig 1, the kinetic model precedes the thermodynamic model. It would be useful to present the models in the order they are cited.

Since we start the model section with the full kinetic model first, we cite Fig. 1B in the introduction of the model section. We use this to demonstrate the simplifications afforded by the thermodynamic approach.

- In the Model section, R should be defined when it’s introduced.
We modified Eqn. 2 to include $R=N_{TF} / K_D^{TF}$.

- In the second paragraph of the section on the phase space approach: “As beta becomes larger, FCmax simply becomes alpha*(P+1)/P”.

Thanks for pointing it out, we fixed the typo.

- The way the main text references Figure 2 is inconsistent with how it’s laid out. The text refers to the left and right panels, but the figure has them labeled as B and C.
We have replaced the left and right with Fig. 2B and 2C.

- In page 4, the authors state that "all results that follow will be derived from the full kinetic model", and then proceed to derive results from the thermodynamic model. More clarity on the model used would help.

We could only get an expression for alpha critical by setting the derivative of fold-change from the thermodynamic model with respect to \beta to zero. This is now mentioned in the manuscript. We did refer to a comparison of alpha_c from the thermodynamic model and full kinetic model (obtained numerically).

- In page 4, the authors discuss that the thermodynamic model applies in the limit where the transcription rate is much slower than RNAP unbinding. Is that generally true? I would expect it to be, but a discussion on realistic parameters would help.

We have now added an explanation
"This is intuitive because in the thermodynamic model, there is no transition of states due to initiation of transcription and the total gene expression is found by summing the product of equilibrium probabilities of different states with their corresponding transcription rate. As such in the full kinetic model, when the transcription rate is very slow, the transition between states become rare events."

- In page 5: "where a critical value of acceleration alpha_c exists".
Changed stabilization to acceleration.

- In Fig 3D-F and Fig 4DE, it would be helpful to show what is the resulting equilibrium R when auto-regulation is introduced. Especially when the phase diagram differs in the two scenarios.
For auto-regulated genes, the fold-change is the representative of the equilibrium R (multiplied by the constitutive expression). So, the heatmap of equilibrium R would be exactly identical to the heatmap of fold-change.

- In Fig 3F, it would be helpful to delineate the parameter space according to the different regions (perhaps a dashed line for beta=1?).

We have added a dashed line that delineates the spaces that would have been separated by beta=1 and removed the beta = 1 line. Hopefully this is now more clear and consistent with previous parameter plots.

- In Fig 3F, FC is no longer monotonic in P. The authors should comment on what is the cause of that.

In this case, there is an interesting relationship because TF number is not held constant. When P is large, there is enough TF in the system that the fold-change will be saturated and thus we expect the same exact qualitative behavior seen in Fig. 2D. (Stabilizing TFs have reduced FC on stronger promoters, and destabilizing TFs have increased FC on stronger promoters). However, in the case of auto-regulation, P-> is defined as FC->1, that is to say, if you don't make TF you don't have regulation. This sets the system apart. So we see this non-monotonic behavior where these two facts clash. For example, in the red and yellow section, where we have a stabilizing activator, the Fold-change must be decreasing at high P, but increasing at low P and we get an intermediate maxima in between. A similar phenomena is seen in the purple and green regions, but instead with a minima. We have now added a explanation of this effect in the main text section.

- Fig 4E seems to suggest that, in certain parameter ranges, response time is less sensitive to parameter alpha than in the case of a TF that doesn't regulate itself. For example, the curves of response time vs FC for

alpha>1 are practically on top of each other. A discussion on how this is achieved by self-regulation would be helpful. Also, those curves should have their gaps fixed.

The gaps in the curves represent parts of the curve where FC is ~1. When the change in expression from the TF gets so close to 0, it becomes impossible to get a meaningful response time. We have added an explanation for the reason for these gaps in the figure legend.

- Technically, dilution of cell components is due to cell growth, not division.

This is a good point. We sometimes think from a number per cell perspective, but number per volume is probably more clear and correct for this framework. We have corrected this language.

- In the section about noise, why vary k_TFon as a proxy for TF number?

The confusion stems from sloppy labeling on our part where we used k_on,TF as both the net, macroscopic binding rate of any TF to the binding site (which scales with TF concentration) and using it as the individual binding rate of a single TF for the site (which does not scale with TF concentration). We now use k_on,TF only to refer to this single TF binding rate and have changed any of the previous uses to N_TF times k_on,TF. Hopefully this clears up the confusion.

- In "Noise in expression," consider including a brief definition/explanation of the Fano factor, as readers may be less familiar with it.

To help clarify exactly what is meant by these terms that quantify noise. We now have added a definition of CV as the ratio of standard deviation to mean and Fano factor as the ratio of the variance to mean at appropriate places.

- Overall, I am confused about the magenta regions on Fig 5. It looks very similar to the blue regions, and I don't quite understand what is the basis for the distinction. An explanation on why the FC in CV shows both local maxima and minima would be helpful.

The blue regions represent regulatory parameter choices where the noise (CV) is monotonically changing with Kon (it monotonically increases for repression and decreases for activation). For the red curves there is a local maximum in CV at a specific Kon which we again see for both activation and repression. The boundary between these two regimes, which we have labeled as purple, actually has a local minimum and maximum in the CV (the derivative of CV as a function of Kon is 0 at two different finite values of Kon. This behavior we only see for repressing interactions. The actual "why" is unclear but we felt we should point out the phenomena since there is something "extra" we see. However, if the extra classification is causing confusion, we could just color them red since they do contain an internal maximum.

- In the conclusion section, a reference to an "AND logic gate" to refer to response times sounds confusing to me.

We have clarified this point in the following way: "For simple regulation, the heat map of response times resembles an ``AND'' logic gate, where the response time is relatively insensitive to regulatory mode unless

the TF acts through both stabilization and deceleration in which case the response of the gene will be slow compared to all other combinations of regulatory mechanisms.”
